# CRISPR/Cas9 Immune System as a Tool for Genome Engineering

**DOI:** 10.1007/s00005-016-0427-5

**Published:** 2016-10-03

**Authors:** Magdalena Hryhorowicz, Daniel Lipiński, Joanna Zeyland, Ryszard Słomski

**Affiliations:** 1 Department of Biochemistry and Biotechnology, Poznań University of Life Sciences, Poznań, Poland; 2 Institute of Human Genetics, Polish Academy of Sciences, Poznań, Poland

**Keywords:** CRISPR/Cas9, Genome editing, Off-target effect, Guide RNA

## Abstract

CRISPR/Cas (clustered regularly interspaced short palindromic repeats/CRISPR-associated) adaptive immune systems constitute a bacterial defence against invading nucleic acids derived from bacteriophages or plasmids. This prokaryotic system was adapted in molecular biology and became one of the most powerful and versatile platforms for genome engineering. CRISPR/Cas9 is a simple and rapid tool which enables the efficient modification of endogenous genes in various species and cell types. Moreover, a modified version of the CRISPR/Cas9 system with transcriptional repressors or activators allows robust transcription repression or activation of target genes. The simplicity of CRISPR/Cas9 has resulted in the widespread use of this technology in many fields, including basic research, biotechnology and biomedicine.

## Introduction

Bacteria and archaea have devised various defence strategies that allow them to survive exposure to foreign nucleic acids such as viral genomes and plasmids. These resistance mechanisms include: prevention of phage adsorption, blocking of phage DNA injection, phage abortive infection systems and restriction modification systems (Chibani-Chennoufi et al. 2004; Chopin et al. 2005; Forde and Fitzgerald 1999). This defensive repertoire has been expanded by the recent discovery of the adaptive microbial immune system, based on clustered regularly interspaced short palindromic repeats (CRISPR) and Cas (CRISPR-associated) genes. CRISPRs were first found in the *Escherichia coli* genome in 1987, when Ishino et al. (1987) discovered loci containing repeat sequences with an unknown function downstream from the *iap* gene. The CRISPR loci are observed in nearly 40 % genomes of sequenced bacteria and nearly 90 % genomes of sequenced archaea (Sorek et al. 2008). Barrangou et al. (2007) demonstrated that CRISPR, together with the associated Cas genes, form an adaptive immunity, which provides resistance against bacteriophage infection. The CRISPR/Cas system is a highly adaptive and heritable resistance mechanism that incorporates short sequences from viruses and other mobile genetic elements into the host’s CRISPR locus to be transcribed and processed into small RNAs that guide the destruction of invading nucleic acids (Charpentier and Marraffini 2014).

## Functioning of the Type II CRISPR/Cas System in Bacteria

**Fig. 1:**
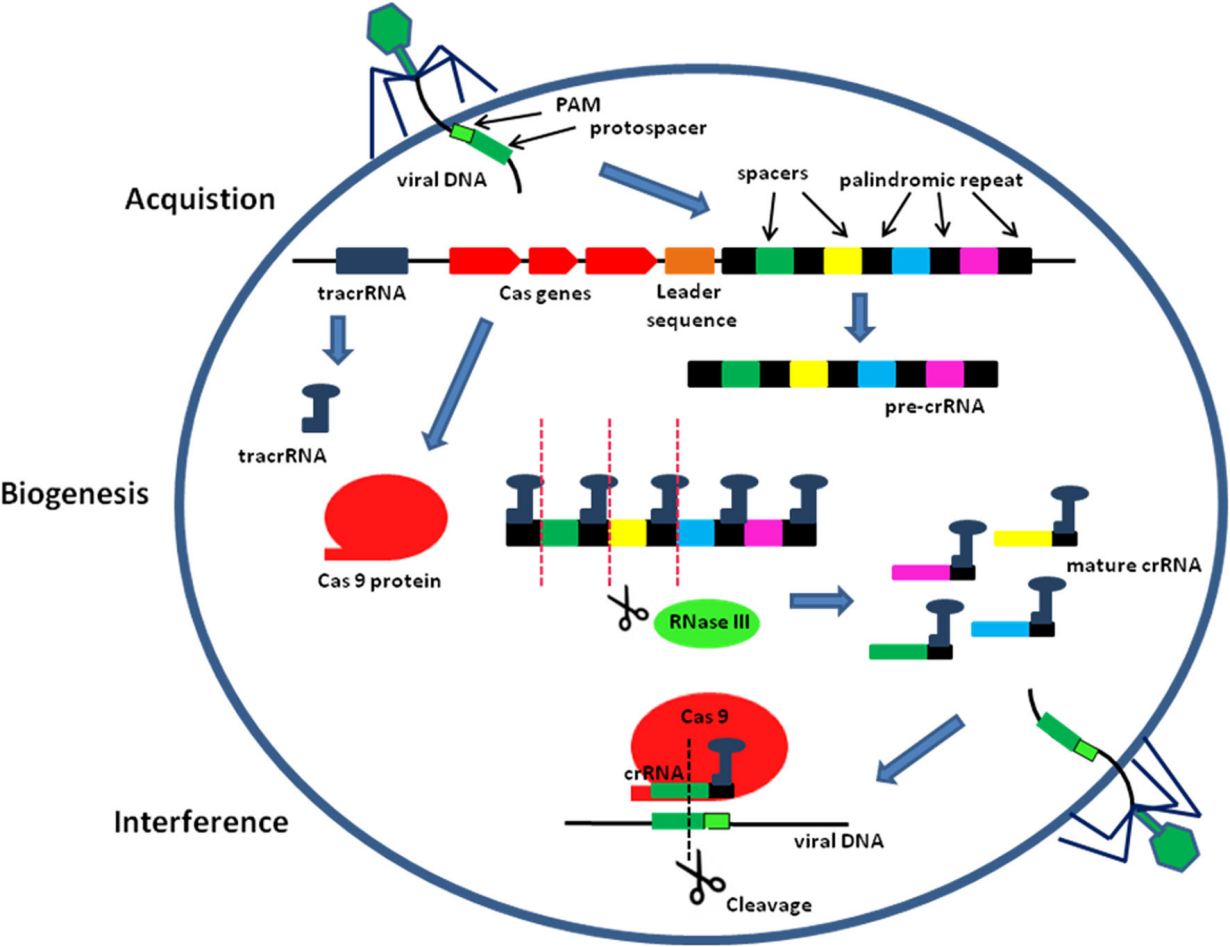
The three stages of the CRISPR/Cas bacterial adaptive immune system: acquisition, crRNA biogenesis and interference of viral DNA

CRISPR/Cas systems of bacterial adaptive immunity are classified into three types according to the differences between the sequence and the structure of Cas proteins. The mechanisms of immunity in types I and III CRISPR/Cas systems are quite complex and are not applied in genome engineering. The simplest among CRISPR/Cas systems is type II, which to interfere with invading genetic elements requires only a single multi-functional Cas9 protein (Makarova et al. 2011). In the endogenous CRISPR/Cas9 system three components are necessary for target cutting: Cas9 protein, CRISPR RNA (crRNA) and transactivating crRNA (tracrRNA), which contributes to crRNA maturation and the formation of the Cas9 complex. The type II CRISPR/Cas system comprises three stages: first, acquisition of CRISPRs; second, crRNA biogenesis; third, interference with invading DNA (Fig. 1). In the acquisition stage, the invading phage DNA is processed by a Cas nuclease into small DNA fragments, called protospacer sequences, and then incorporated into the CRISPR locus of the bacterial genome as a new spacer (Wiedenheft et al. 2012). Each CRISPR array encodes acquired spacers that are separated by repeat sequences. The selection of protospacers depends in part on the specific recognition of protospacer adjacent motifs (PAMs) present within the viral genome. However, protospacer sequences incorporated into the CRISPR locus do not contain PAM sites (Mojica et al. 2009). The identity of the PAM sequence depends on the species of the Cas9 protein (for example: 5′NGG-3′ PAM in *Streptococcus pyogenes*, 5′-NGGNG-3′ PAM in *Streptococcus thermophilus* and 5′NNNNGATT-3′ PAM from *Neisseria meningitides*) (Cho et al. 2013; Hou et al. 2013; Karvelis et al. 2013). Subsequently, in the biogenesis step, the CRISPR locus is transcribed into a long precursor CRISPR RNA (pre-crRNA). TracrRNA hybridizes to the repeat sequences of the pre-crRNA and then endogenous RNase III cleaves this complex, yielding mature crRNAs, each containing one spacer and partial repeat sequence (Deltcheva et al. 2011; Pougach et al. 2010). Finally, in the interference step, mature crRNA guides Cas9 protein to the complementary foreign nucleic acids, triggering degradation of the DNA sequences of invading phages (Garneau et al. 2010; Marraffini and Sontheimer 2008). The Cas9 protein contains HNH (named for characteristic histidine and asparagine residues) and RuvC (named for an *E. coli* protein involved in DNA repair) nuclease domains, which cleave the DNA complementary strand and the non-complementary strand, respectively (Jinek et al. 2012). This endonuclease cleaves the viral genome by introducing double-strand breaks (DSB) 3 bp upstream of the appropriate PAM. The PAM sequence is crucial for the interference stage because it enables one to distinguish between the invading foreign DNA and the CRISPR loci in the host genome, which do not contain PAM (Shah et al. 2013).

## Exploiting CRISPR/Cas9 Systems for Genome Engineering

**Fig. 2:**
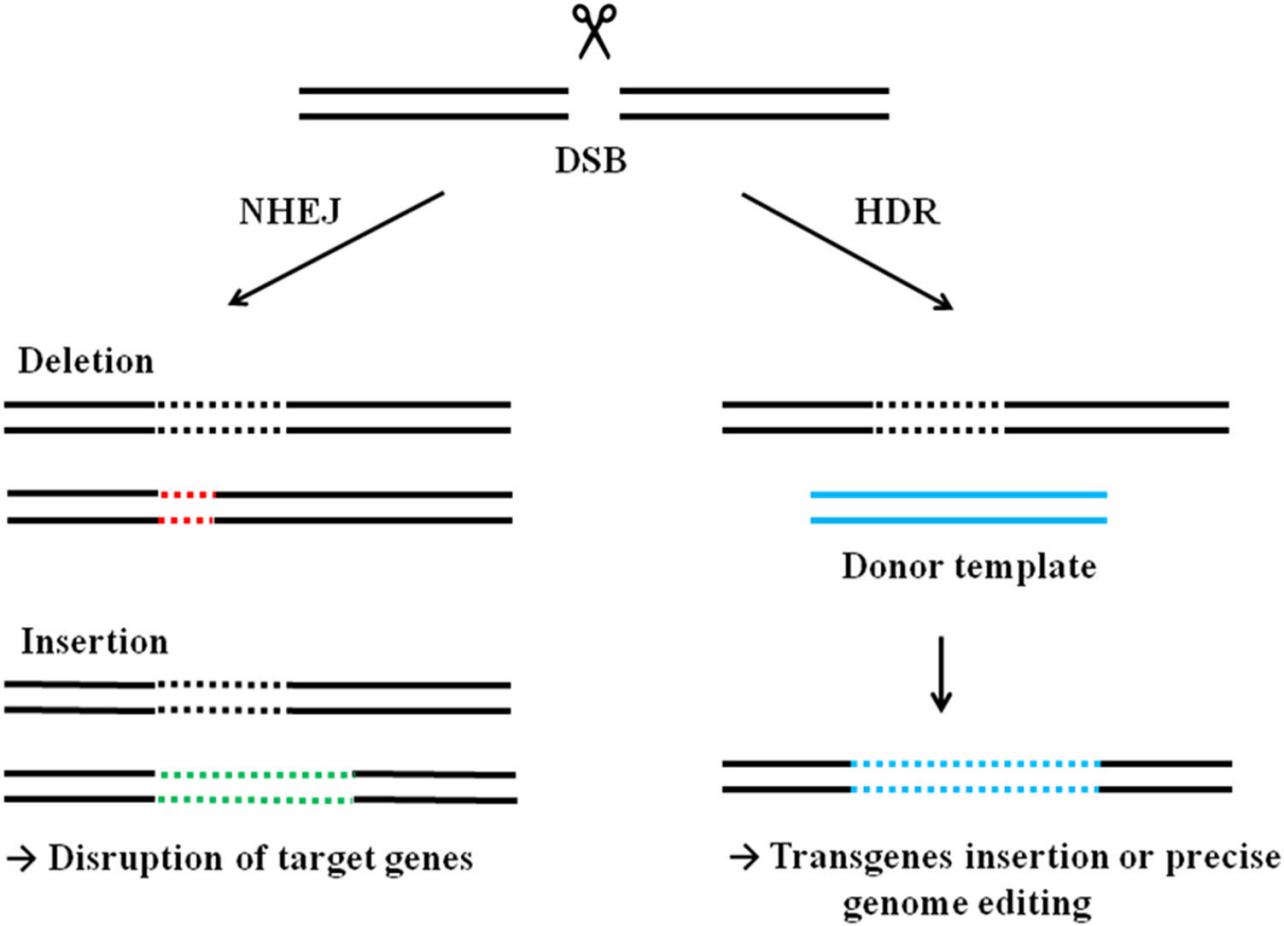
Engineered nuclease-induced genome editing. A double-stranded break (DSB) in the targeted sequence can be repaired through nonhomologous end joining (NHEJ) or homology-directed repair (HDR) pathways

The guide sequence within CRISPR spacers typically corresponds to foreign viral genomes constituting the form of the acquired immunity of bacteria, but can easily be substituted by a sequence of interest to target the Cas9 protein. In 2012, two research groups published findings stating that purified Cas9, derived from *Streptococcus thermophilus* or *Streptococcus pyogenes*, can be guided by crRNAs to cleave target DNA in vitro (Gasiunas et al. 2012; Jinek et al. 2012). Moreover, the RNA components of the CRISPR/Cas9 system can be used separately as crRNA containing the targeting guide sequence and constant tracrRNA molecules, or as single guide RNA (sgRNA) chimera, consisting of a fusion of a crRNA and a tracrRNA facilitates rapid implementation of the CRISPR/Cas9 system for genome engineering. Cas9 target recognition requires both the PAM sequence in the target DNA and RNA–DNA complementarity base pairing between the 20-nt guide RNA sequence and the complementary target DNA sequence (Jinek et al. 2012). Cas9-generated site-specific DNA double-strand breaks induce endogenous cellular DNA repair processes, which can be exploited to engineer the genome. DSBs are generally repaired by one of two pathways, homologous directed repair (HDR) if the homologous template is available or otherwise by nonhomologous end joining (NHEJ). NHEJ is an error-prone process that can rapidly ligate the broken ends but generate small insertions and deletions (indels) at targeted sites, which often result in the function of target genes being disrupted or abolished. Alternatively, DSB may also be repaired via HDR, which is able to recombine exogenous DNA, and can be used to introduce transgenes or precise genome editing (Fig. 2).

Earlier technologies to introduce DSBs, such as zinc-finger nucleases (ZFN) and transcription activator-like effector nucleases (TALEN), are fusions of the nonspecific DNA cleavage domain from the FokI restriction endonuclease and sequence-specific DNA binding domains derived from zinc-finger and TALE proteins. ZFNs and TALENs require recoding of proteins for each new target site, which is time consuming and very expensive. In contrast, CRISPR/Cas9 to target a new site only requires a suitable sgRNA to be designed, because the Cas9 protein remains the same in all cases. Moreover, the expression of Cas9 and multiple guide RNAs can be used for simultaneous editing of several target sites in the mammalian genome (Cong et al. 2013). The fact is that CRISPR/Cas9 technology is easy to design and produce, is highly efficient and inexpensive. Exemplary protocols for preparation of CRISPR/Cas reagent to create genetically modified mouse were described by Harms et al. (2014).

## Non-nuclease Uses of Cas9 Protein

The CRISPR/Cas9 system is a powerful tool for genome modification but can also be used to regulate the expression of endogenous genes (Qi et al. 2013). Catalytically dead Cas9 (dCas9) protein with inactive RuvC and HNH nuclease domains retains the ability to bind to target DNA (complementary to the sgRNA) and causes repression of the target gene by a steric block that stops transcript elongation by RNA polymerase. This CRISPR-based interference (CRISPRi) is a similar approach to RNA interference (RNAi) with the difference that CRISPRi regulates gene expression on the transcriptional level, while RNAi on the mRNA level. Moreover, dCas9 protein can be fused to a transcriptional activator (e.g., VP64) or to transcriptional repressors (e.g., Krüppel associated box). These dCas9 fusion proteins are targeted to the indicated promoter region, resulting in transcription repression or activation of target genes (Gilbert et al. 2013; Maeder et al. 2013). Significantly, this does not permanently modify the genome because dCas9-mediated gene activation or repression is reversible. The fusion of activator or repressor to dCas9 can be used for studying of specific genes and identify the functions of new genes (Konermann et al. 2015). It has been also demonstrated that an EGFP-tagged dCas9 protein can be used to locate and visualize specific genomic loci in living cells (Chen et al. 2013).

## Off-Target Effects

The CRISPR/Cas9 system enables permanent genome modification and, therefore, its off-target effects, which are alterations occurring outside the targeted locus, should be controlled. The off-target sites are identical or highly homologous to the target DNA sequence and can be recognized by Cas9 protein, which tolerates some mismatches between the sgRNA and the target DNA (Hsu et al. 2013). In general, the mismatches closer to the 5′ end of the 20 nt targeting region of the gRNA are better tolerated than mismatches close to the PAM at the 3′ end. Interestingly, some studies suggest that even Cas9 which is able to bind to off-target sites cleaves only some of them (Wu et al. 2014). Indel mutation at the off-target sites can be detected by searching the sequences with high similarity to the target locus and genome-wide identification of Cas9 cleavage profile by GUIDE-seq or by whole genome sequencing (Smith et al. 2014; Tsai et al. 2014b).

Although there are some studies demonstrated low incidence of off-target mutations in Cas9-modified mice and Cas9-engineered human pluripotent stem cells (Iyer et al. 2015; Veres et al. 2014), various approaches have been explored to reduce the off-target effects. First, when gRNAs are designed, it is recommended to select those target sites predicted to have the fewest off-target sequences. Several online tools are available for facilitating the identification of suitable guide sequences (e.g., http://crisp.mit.edu/) and that web-based software supplies the specificity score of off-target effects. Generally, use of a high-score guide yields higher target specificity with a lower chance of off-target mutagenesis. Additionally, it has recently been suggested that off-target effects can be reduced using guide RNA truncated by 2–3 nt at the 5′ end. These truncated gRNAs may increase specificity because a shorter sgRNA sequence has a decreased mismatch tolerance, although this manipulation results in a reduction in the absolute efficiency of on-target genome editing (Fu et al. 2014).

Another way of limiting off-target endonuclease effect is to use a Cas9 nickase mutant and pairs of gRNAs to introduce targeted double-strand breaks (Cho et al. 2014; Shen et al. 2014). A mutant form of Cas9 introduces a single-stranded break (called a nick) by catalytically inactivating the RuvC or HNH nuclease domains (Jinek et al. 2012). In this method, pairs of Cas9 nickases are targeted to generate two nicks close to each other on opposite strands of the genomic target DNA, which can be the equivalent of a DSB. The double nicking system can significantly increase specificity because off-target single nick should be repaired immediately without any undesirable mutation. Furthermore, the chance that two nicks occur together in the same arrangement like target sites elsewhere in the genome and generate DSB is extremely small. Importantly, using Cas9 nickases significantly reduces off-target mutagenesis but at the expense of lower efficiency (Li et al. 2014).

Similarly, fusions of catalytically inactive Cas9 (dCas9) with FokI nuclease domain can also improve DNA cleavage specificity (Guilinger et al. 2014; Tsai et al. 2014a). In this approach, designing two gRNAs, which are bound in close proximity to two unique target sites, are necessary. Binding dCas9-FokI fusion proteins to the target sequences and dimerization of pair FokI domains is required to generate a DSB. This system is regarded to have even lower off-target effects than dual nickase because monomeric FokI nuclease domains are not catalytically competent (Guilinger et al. 2014).

The dosage of CRISPR/Cas9 components is also an important factor affecting off-target mutagenesis and should be carefully monitored. As previously mentioned, Cas9 can tolerate some mismatches within the target site leading to off-target activity. It has been proven that mismatches appear to be better tolerated when Cas9 occurs in high concentrations. Therefore, decreasing the amount of Cas9 in the CRISPR/Cas9 delivery system substantially reduces off-target effects. Unfortunately, the efficiency of on-target cleavage is also at a lower level (Hsu et al. 2013).

The latest approaches to decrease off-target effects of CRISPR/Cas9 involve new Cas9 variants: eSpCas9 and SpCas9-HF1. Slaymaker et al. (2016) demonstrated, through structure-guided protein engineering, that neutralization of positive charges in the HNH/RuvC groove can decrease off-target indel formation while maintaining on-target activity. Electropositivity reduction of the HNH/RuvC non-target strand groove weakens the interactions between groove and the negatively charged DNA, therefore, destabilizes strand separation and decreases the Cas9 nuclease activity. To neutralize the positively charged groove, the authors generated a variety of alanine substitutions within the groove at Cas9 mutants. The eSpCas9(1.1) mutant revealed decreased genome-wide off-target effects and did not cause any new off-target sites. Another way to enhance CRISPR targeting specificity with engineering Cas9 has been described by Kleinstiver et al. (2016). The scientists disrupted interactions between Cas9 protein and the phosphate backbone of the target DNA via mutations at four amino acid residues (N497A, R661A, Q695A, and Q926A). The mutant SpCas9-HF1 (SpCas9 high-fidelity variant number 1) had on-target activities comparable to wild-type SpCas9 and reduced off-target cuts to an undetectable level.

## Application of CRISPR/Cas9 System

The type II CRISPR/Cas system has been rapidly and widely utilized to target genome modifications in various species and cell types, including plants (Jiang et al. 2013), insects (Bassett et al. 2013), mice (Seruggia et al. 2015), rabbits (Honda et al. 2015), pigs (Whitworth et al. 2014), monkeys (Niu et al. 2014) and human cells (Liang et al. 2015). CRISPR/Cas9 is an efficient system for genome engineering of animals, which gives unlimited possibilities for xenotransplantation, regenerative medicine and using animals as models for studying of human diseases. Genetically modified large animal models are of increasing significance in biomedical research. To obtain large transgenic animals with CRISPR/Cas9 system, plasmids expressing Cas9 and properly designed sgRNA can be easily introduced into the cells by transfection and used in somatic cell nuclear transfer (Ni et al. 2014). Another route relied on in vitro transcription of Cas9 and sgRNA and direct injection of Cas9 mRNA and sgRNA into fertilized zygotes (Whitworth et al. 2014). CRISPR/Cas9 system enables multiplex genome editing. Wang et al. (2013) demonstrated that CRISPR/Cas allowed the simultaneous disruption of five genes in mouse embryonic stem cells with high efficiency. Moreover, the application of CRISPR/Cas9 multiplexability to inactivate porcine endogenous retrovirus (PERVs) in a swine kidney epithelial cell line has recently been described. In this study, two sgRNAs were designed to disrupt 62 copies of the PERV pol gene critical for retroviral activity. The scientists noted a >1000-fold reduction in PERV transmission to human cells, which suggests the possibility to inactivate PERVs for clinical application of porcine-to-human xenotransplantation (Yang et al. 2015).

Mutagenic chain reaction (MCR), described by Gantz and Bier, is another example of application of the CRISP Cas9 system. MCR based on the CRISPR/Cas9 system is used for generating autocatalytic mutations to generate homozygous loss-of-function mutations (Gantz and Bier 2015). The authors developed a technology to convert a *Drosophila* heterozygous recessive mutation into homozygosity. MCR’s construct consist Cas9 gene and gRNA which are flanked by two homology arms targeting the genomic sequences to be cut. Usually, mutations carried on one of a pair of chromosomes are inherited by only half the offspring. MCR system allows a mutation on one chromosome to copy itself in both somatic and germline cells “gene drive”, which results in that almost all offspring will inherit the change. MCR could be used to eliminate diseases such as malaria, yellow fever and others by altering insect species to eradicate invasive species and to pave the way toward sustainable agriculture by reversing pesticide and herbicide resistance. Despite the fact that this technology can be very useful, there are concerns about the risks associated with release of MCR organisms into the environment because modifying a whole population, or eliminating it altogether, could have unknown consequences for an ecosystem.

The CRISPR/Cas9 system can also be used as a therapeutic technology for treating genetic disorders such as Duchenne muscular dystrophy (DMD) by correcting the dystrophin gene mutation (Long et al. 2014) or cystic fibrosis by repairing the mutation in the CFTR gene (Schwank et al. 2013). DMD is caused by mutation in the dystrophin gene, which consists of 79 exons. Removing one or more exons from the mutated transcript by CRISPR/Cas9 system allowed for production of truncated, but still functional dystrophin protein in a mouse model of muscular dystrophy (Tabebordbar et al. 2016). Moreover, restored dystrophin protein expression was obtained by exon skipping, frameshifting, and exon knock-in in DMD-patient-derived induced pluripotent stem cells. The exon knock-in was the most effective approach and resulted in restoration of the full-length dystrophin protein (Li et al. 2015).

Additionally, the CRISPR/Cas9 system has therapeutic potential for preventing coronary heart disease. PCSK9 (proprotein convertase subtilisin/kexin type 9) is a protein expressed in liver cells. It has been shown that spontaneous loss-of-function PCSK9 mutations reduced low-density lipoprotein cholesterol levels (Cohen et al. 2005). Thus, CRISPR/Cas9 technology was used for generating a knockout of PCSK9 gene in mice. Genome editing with CRISPR/Cas9 successfully and efficiently disrupted the PCSK9 gene in vivo, leading to reduced plasma cholesterol levels. It may have therapeutic potential for the prevention of cardiovascular disease in humans (Ding et al. 2014). Another example of the application of the CRISPR/Cas9 system is in the treatment of viral infections. For example, CRISPR/Cas9 can efficiently knockout the CCR5 (CC chemokine receptor 5) gene to prevent HIV-1 integration (Ye et al. 2014). In turn, another group demonstrated that the CRISPR system targeted at the surface antigen (HBsAg)-encoding region of HBV efficiently produced mutations in HBV DNA. This resulted in inhibition of HBV replication and expression, and can be used as a new therapeutic strategy for HBV infection (Zhen et al. 2015).

## Conclusion

In August last year, the CRISPR/Cas as an Adaptive Bacterial Immune System on Its Way to Become a Game Changer in Genetic Engineering was one of the topics of the Biological Weapons Convention Meeting of Experts of United Nations, Geneva. The reason was the emergence of reports about the possibilities of dual use of this technology. Presentation concerning CRISPR/Cas9 technology was met with great interest among the delegations of individual countries but also raised many concerns. Unease was raised by articles pointing at the possibility of using this technology to induce cancer in mice to create model for human lung cancer (Maddalo et al. 2014). Components of CRISPR/Cas9 delivered to mice by inhalation using adenovirus easily results in the formation of lung tumours in these animals already after a few weeks. Greater concern is how easily and quickly one can induce tumours in different tissues and organisms using this technology and what kind of consequences will be associated with the improper designing or using of CRISPR/Cas9 system. Some researchers even call for an end to work using CRISPR/Cas9 technology but the experience of authors, in particular a leader of the team (prof. R. Słomski), shows that in the history of genome engineering in 1974 Paul Berg and other scientists in the field of recombinant DNA drafted a letter calling upon “scientists throughout the world” to suspend certain types of studies until hazards could be assessed (Berg et al. 1974). However, despite a number of concerns they have failed to stop them with the perspective of time contributed to new discoveries and achievements in the field of biology, agriculture and medicine.

The CRISPR/Cas9 is an efficient, cheap and easy-to-use tool for a genome editing, which is rapidly being applied to many fields, including generation of the animal models, functional genomic screening and correction of genetic disorders. However, this technology must be used carefully. Planning CRISPR/Cas experiments, precise design of gRNA, choosing the best variant of Cas9 and genome-wide searching of potential off-target sites should be taken into account. Scientists should think cautiously about how they are going to use that powerful technology. Only then, it will be possible to use the CRISPR/Cas9 system safely.
